# Development of Alternative Screw Concepts for Rubber Extrusion Based on Simulative and Experimental Methods

**DOI:** 10.3390/polym18091085

**Published:** 2026-04-29

**Authors:** Leon Schmidt, Volker Schöppner

**Affiliations:** Kunststofftechnik Paderborn, Paderborn University, 33098 Paderborn, Germany

**Keywords:** rubber, simulation, CFD, extrusion, screw design, alternative screws, pinless rubber extrusion

## Abstract

This article deals with the substitution of a pin-type rubber extruder by alternative screw concepts based on circumferential and axial flight offsets. Alternative screw concepts were investigated using simulative and experimental methods. This revealed the opportunities and risks of the simulations and the suitability of pinless concepts for rubber extrusion. Good functionality of the simulations is shown for an SBR rubber mixture, except for thermal properties. For the SBR mixture used, pinless designs can achieve comparable material and thermal homogeneity to conventional pin extruders while achieving up to 10% higher throughputs. Therefore, this study shows that these alternative designs can increase the cost-effectiveness of rubber extrusion and be designed using simulative methods.

## 1. Introduction

The processing of rubber on extruders has been an important branch of industry for many years. Technical elastomer products, tires, and everyday objects such as rubber bands are an integral part of our daily lives. In 2024, 30.9 million tons of natural rubber and synthetic rubber were produced worldwide. This represents the highest production volume to date in the 21st century [1]. Nevertheless, due to elevated energy and raw material expenses [2], cost-effectiveness is becoming increasingly important for rubber extrusion.

Two different extruder concepts are used for processing, which differ in the initial rubber temperature, which can be cold or preheated [3]. Hot-fed extruders are fed with heated rubber in an upstream step so that the extruder takes over the homogenization of the material and builds up the necessary pressure to overcome the back pressure [4]. Therefore, hot-fed extruders can be kept relatively short. In practice, however, they play a rather subordinate role. Cold-fed extruders, on the other hand, are fed with rubber strips at ambient temperature. These must then be heated and converted into a homogeneous mass, which needs to be achieved by the rubber extruder. The extruder, therefore, pulls in the rubber stripes by a feed roller and conveys the material to the die by a rotating screw while concurrently building up pressure. The homogenization is achieved by dividing and reuniting the material stream and crossflow effects in the channels of the screw. As a result, cold-fed extruders have a much greater length than warm-fed extruders, which do not need to thermally homogenize the material. Nevertheless, cold-fed extruders are mostly used in today’s industry in connection with pins because this combination leads to remarkable material qualities and reduces the required space and investment costs, as a separate rolling mill is no longer needed for preheating [4,5,6,7]. In addition to excellent thermal and material homogeneity, the cold-fed pin extruders also ensure a highly robust process, thereby enabling the production of products of consistent quality at reasonable costs [5,6].

In contrast to thermoplastic extruders, there is no classic melting of the polymer when processing the rubber. The rubber is merely heated, which reduces its viscosity and improves its processing properties, but no phase transformation takes place. Another decisive difference in the processing of many other plastics on extruders is the material itself. Rubber compounds generally have very high viscosities, which require special plant technology. Rubber extruders are usually equipped with significantly more powerful drives. In addition, the rubber is much more difficult to homogenize due to its high viscosity and elasticity. This is shown by the appearance of a so-called cold core in the middle of the channel [8]. The comparatively deep channels of the rubber screws, in combination with the high viscosity, only allow the cold core to be reduced through good distributive mixing. For this purpose, pins protruding into the flow channel are regarded as state-of-the-art. In the majority of cold-fed rubber extruders, they are currently essential for attaining good extrudate quality [4]. By continuously dividing and recombining the rubber melt within the screw channel, they effectively disrupt the block flow present in the cold core. This promotes efficient distributive mixing and, consequently, enhances both the thermal and material homogeneity [5,9]. Another special feature of rubber extruders is the water temperature control. Due to the low temperature level but the high heating and cooling capacity required, both the barrel elements and the screw are generally temperature-controlled using water.

### 1.1. Quality Criteria of Extruded Rubbers and Rubber Extruders

The quality of extruded rubber compounds can be assessed based on various criteria. The primary criterion in practice is compliance with the product geometry while maintaining homogeneous properties of the product across its cross-section [10]. However, this is based on the prerequisite that sufficient thermal and material homogeneity of the rubber is achieved [11]. These criteria are used to assess the extrudate but also to provide information about the suitability of the extruder for the selected process. Another important criterion is the efficiency of the extruder, which is mainly influenced by the throughput achieved.

#### 1.1.1. Thermal Homogeneity

Thermal homogeneity represents how evenly the temperature is distributed across the channel cross-section of the extruder or the cross-section of the extrudate. The more evenly the temperature is distributed, the higher the resulting thermal homogeneity. In reality, the temperature is never totally even across all areas of the extrudate. Thermal influences on the material are not only caused by the heating or cooling through the barrel or screw. The extrusion of the material introduces shear into the material. This, in turn, leads to a local increase in temperature due to dissipation, as the shear is also not introduced evenly into the material. The mixing effects, which should generally lead to a reduction in temperature differences, also lead to a certain dissipative heat input, which influences the thermal homogeneity. This combination means that the optimum state cannot be achieved, but a good approximation can be achieved if a good thermal mixing quality is ensured by the extruder. The thermal mixing quality can be quantified if temperatures at the screw tip are available. The maximum temperature difference in the channel cross-section at the screw tip can be considered; the greater this is, the lower the thermal homogeneity [12]. The final assessment should be carried out at the tip of the screw, as all the influences introduced by the extrusion are considered.

The temperature of the extrudate is currently kept relatively cold—but definitely below the critical temperature of the used material [13]—to avoid any scorching effects, as the maximum temperature is substantially higher than the average due to peaks resulting from shear warming connected with insufficient mixing and low thermal conductivity of the rubber [5]. Good thermal homogenization reduces those peaks in temperature and allows higher temperatures of the extrudate, which could save energy during the vulcanization process, as less thermal energy needs to be introduced into the material.

#### 1.1.2. Material Homogeneity

The material mixing quality represents how well the rubber compound is mixed in the extruder across the channel cross-section. In the processing industry, usually only one continuous strip is added, which already contains all the components and has been premixed. Yet good material mixing is also necessary here, as agglomerates are common in the premixed material. For a high-quality product, it is essential that all components of the rubber mixture, which usually consists of ten or more ingredients, are evenly distributed in the product so that it has identical properties at every position. However, material homogeneity can only be characterized with considerable effort compared to thermal homogeneity. One possibility is to add individual particles and evaluate their distribution. In that case, it must be ensured that the selected particles influence the flow as little as possible and therefore do not falsify the results. Another possibility for experimental investigations is the addition of rubber in different colors. This allows the mixed proportion of the cross-sectional area of the emerging extrudate to be evaluated [12,14]. The mixing effects are also subdivided into different effects. On the one hand, they can be divided according to the direction of mixing. There is so-called transverse mixing, which takes place across the cross-sectional area of the extruder and is of great importance for the material mixing quality. There is also longitudinal mixing, which is closely related to the residence time distribution and describes the mixing of the rubber in the longitudinal direction of the extruder. However, as the rubber extruder is usually fed with mixtures with predominantly constant proportions, this is of secondary importance. On the other hand, a distinction can be made between distributive and dispersive mixing. Dispersive mixing is the breaking up of agglomerates of individual components [12]. These must be broken up first so that they can then be distributed as evenly as possible across the channel cross-section by the distributive mixing effects. In practice, it is difficult to separate the effects from one another, as they usually occur simultaneously because mixing results from the distribution and reorientation of the flow in the screw channels.

#### 1.1.3. Scorching

The term scorching is used in the rubber industry to describe the cross-linking of small parts of the rubber in the extruder. This occurs when undesirably high temperatures occur locally in the rubber due to an unsuitable temperature profile of the extruder or high dissipative energy. In principle, cross-linking in the extruder causes the rubber to lose its plastic properties to a limited extent and to change to a purely elastic state. This means that the desired shaping in the die is no longer possible, as the material can only be deformed reversibly in some cases and returns to its original shape after exiting the die. Even if only small proportions of the extrudate are affected, this results in inadequate product quality, as the cross-linked areas form inclusions that can weaken the geometry or restrict the function. It is comparatively easy to remedy an inadequate temperature profile as the cause, so that this does not represent a defect in the design of the extruder but is merely due to suboptimal process settings. Excessive dissipative energy input, on the other hand, can possibly be reduced or avoided by adjusting the rotational speed or the barrel and screw temperatures; nevertheless, this also represents a deficiency in the design of the extruder or screw, as a reduction in rotational speed correlates with a reduction in throughput and cooling through the barrel and screw that is usually not sufficient and makes the processing more difficult. Scorching must be avoided in any case, as the product would possibly no longer meet the specifications and its properties would become inhomogeneous and unpredictable [15,16].

#### 1.1.4. Efficiency

An additional point for evaluating an extruder concept is its efficiency. In principle, an extruder should be able to achieve the highest possible throughput, as this is one of the foundations for the economic efficiency of its operation [15]. The throughput correlates with the screw speed, which is why it is either helpful to compare the specific throughput [17] or to find the operation point with the highest possible throughput and compare the absolute values for different concepts. But for the throughput maximization, boundary conditions need to be set that limit, e.g., the maximum temperature of the extrudate. Furthermore, the energy consumption should be considered, as differences in the consumption may lead to high cost impacts that may offset the advantages of higher throughputs.

### 1.2. Disadvantages of Recently Used Concepts

Currently, cold-feed pin extruders are predominantly used as they are characterized by their excellent mixing quality, and a lot of experience has been accumulated over the last few decades among manufacturers and users with regard to the concept. For this reason, adapting the concept for new products is relatively simple and cost-effective compared to developing a different concept. However, it is also known that although the pins have a positive effect on the mixing quality due to their intervention in the flow channel, this also reduces the free channel volume for the melt, which has a negative effect on the throughput. In addition, the pins are a pressure consumer and, therefore, increase the necessary torque and, in conjunction with this, the energy consumption of the extruder [18]. The pins also mean an enormous amount of work in the event of a screw change or for cleaning and maintenance of the system in general, as the screw can only be removed from the extruder when the pins are removed. Depending on the size and degree of pinning of the extruder, the required set-up time and the associated downtime of the system can be doubled. This, in turn, costs the operator money in two ways, as they have to assign personnel to set up and, at the same time, cannot produce on the line and therefore do not earn any money. However, alternative concepts have so far only been considered to a very limited extent, as the development costs are enormous since they are mostly based on experimental trial and error.

### 1.3. Simulation of Extrusion Processes

The simulation of extrusion processes for thermoplastic melts is state-of-the-art and can be carried out by available CFD programs or special simulation programs for extrusion, like the KTP’s Software REX (Ansys Fluent 2023 R1). CFD programs can be used for rubber too, but because of its specialties, like wall-slip effects and elastic effects, the material behavior is usually not depicted completely, which leads to potentially huge deviations between reality and simulation. Nevertheless, previous projects at KTP and other universities have worked on simulations of rubber processing for special issues and achieved good results. However, those simulations were mostly focused on several aspects of the process and therefore used e.g. isothermal conditions [16].

Regarding the quality criteria of simulated rubber, these remain identical to those in reality. Thermal homogeneity can be investigated by the temperatures of single cells, a certain area, or the whole simulated volume. The resulting throughput is calculated by the CFD software according to the geometry and boundary conditions. Material homogeneity, on the other hand, is not determined by different colors but by the simulation of pathlines, which result from the position of massless tracer particles. Their positions in a certain region, e.g., the outlet of the simulation area, are determined. From those positions, a Delaunay triangulation can be performed, which delivers unambiguous triangles whose surfaces and standard deviations can be calculated. The relationship between the average surface and the standard deviation is a good indicator to compare the mixing quality [16,19].

### 1.4. Development of Alternative Screw Concepts for Rubber Extrusion

As already stated at the beginning of this article, the development of alternative screw concepts is approached very cautiously from different sides of the industry, as it is linked to high costs and uncertainties. However, in recent years, there have been a few research projects that have worked on alternative concepts like the projects on the “simulation of mixing elements” or “mixing behavior of rubber extruders” [16], which aimed to achieve comparable extrudate qualities to the state-of-the-art but at lower costs or with higher throughputs. In these projects, different alternative screw geometries were investigated that were based on mixing geometries from thermoplastics extrusion and circumferential and axial flight offsets of the screw. While mixing elements like the pineapple mixer—which were adapted from thermoplastics—were not suitable, as they lead to locally high shear-stresses and therefore temperature peaks, the flight offsets showed good results. The combination of circumferential and axial flight offsets led to comparable thermal and material homogeneity of the extrudate as a pin extruder, but allowed much higher throughputs at only slightly higher torque. Therefore, the project showed that screw concepts based on offsets of the flights seem to be able to replace pin extruders, but it also revealed that the correct geometry is crucial, as circumferential offsets alone led to a lower throughput compared to pin extruders [16].

Furthermore, the concept of flight offsets is already used in the second extruder of tandem extrusion lines for thermoplastic foam extrusion. The screws show similarities to rubber screws as they are used to cool down the thermoplastic melt, which is achieved by deep channels and slotted flights (flight offsets) that mix warmer and colder melt to reach a homogeneous and cooler mass [20].

For that reason, further investigations into flight offsets and their simulation seem necessary to enable the development of adjusted screw concepts for different materials. To improve the understanding of screw designs and the development of alternative screw concepts, this article presents methods for simulating alternative screw designs and demonstrates how these can be applied in practice to an application-specific SBR blend. These simulations enable the significantly more cost-effective design of alternative screw concepts without pinning, which had not been feasible until now due to the recently used cost-intensive trial-and-error process. This opens up the possibility of replacing the pin extruder in SBR extrusion while concurrently improving economic efficiency through up to 10% higher throughput and reduced maintenance and changeover costs.

## 2. Materials and Methods

### 2.1. Materials

For investigating screw concepts with flight offsets, it can be assumed that the results are dependent on the materials used, as the flow behavior of the materials affects the mixing efficiency of the flight offsets. Therefore, the material used here was developed in collaboration with partners from the industry to ensure its practical relevance.

Nevertheless, the mixture does not contain carbon black, as it was needed in two different colors for the evaluation of the material’s mixing quality in experimental investigations using slices from the extrudate and so-called dead stops, as explained in [21]. The SBR rubber mixture is available in blue and yellow with otherwise identical properties.

The SBR compound, presented in Table 1, is based on a tire mixture and on 90 phr SBR and 10 phr NR. The Mooney viscosity ML1 + 4 (100 °C) of this compound is 54 MU. Based on cross-linking isotherms measured at an RPA D-RPA 3000, Montech Prüfmaschinen GmbH, Buchen, Germany, the critical temperature was defined as 180 °C.

The material properties of this SBR compound can be described by the following equations, which were also used for the simulations made throughout the project. The viscosity can be described by the Carreau law connected with a WLF temperature shift, as shown in Equations (1) and (2).

(1) $$\eta =\frac{A*\alpha_{T}}{{\left(1+B*\dot{\gamma }*\alpha_{T}\right)}^{C}}$$

(2) $$\log\left(\alpha_{T}\right)=\frac{8.86*(T_{B}-T_{S})}{101.6\;K+T_{B}-T_{S}}-\frac{8.86*(T-T_{S})}{101.6\;K+T-T_{S}}$$

where

η: Viscosity;
$\alpha_{T}$: Temperature shift;
*A*: Zero-shear viscosity;
*B*: Reciprocal transition shear rate;
*C*: Slope;
*T*: Temperature;
$T_{B}$: Reference temperature;
$T_{S}$: Standard temperature.

For this SBR compound, the components of Equations (1) and (2) are best described by the values shown in Table 2.

The density, thermal conductivity, and heat capacity can be described by Equations (3)–(5) by taking into account a linear temperature dependence and a linear pressure dependence for the density, which showed sufficient accuracy in the temperature and pressure range needed in the simulations.

(3) $$\rho =1.29733+6.63*10^{-10}*p-6.3*10^{-4}*T$$

(4) $$\lambda =0.14236+1.8*10^{-4}*T$$

(5) $$c_{p}=0.32919+3.47*10^{-3}*T$$

where

ρ: Density;
λ: Thermal conductivity;
$c_{p}$: Heat capacity.

### 2.2. Methods

**Figure 1:**
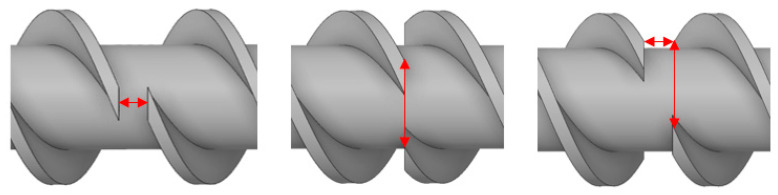
A 10 mm axial offset (**left**), 60° circumferential offset (**middle**), and combination of 10 mm axial and 60° circumferential offset (**right**).

This paper includes both simulative and experimental investigations, taking into account different screws with circumferential and axial offsets. In the following sections, circumferential and axial offsets are defined, as shown in Figure 1.

Two screws were already available at the Kunststofftechnik Paderborn (KTP), with geometries as shown in Table 3. Both screws have a diameter of 60 mm, with a total length of 18.3 D and a channel depth of 12 mm. They both have two flights, but screw A can be equipped with pins, while screw B does not use pins. Some experimental investigations of these screws were already presented in [21].

**Figure 2:**
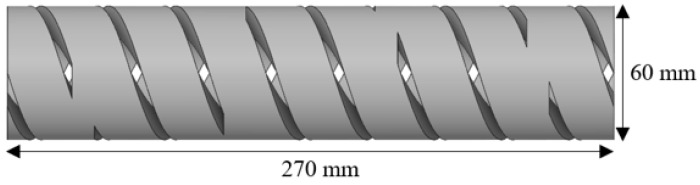
Flow channel for simulations.

**Figure 3:**
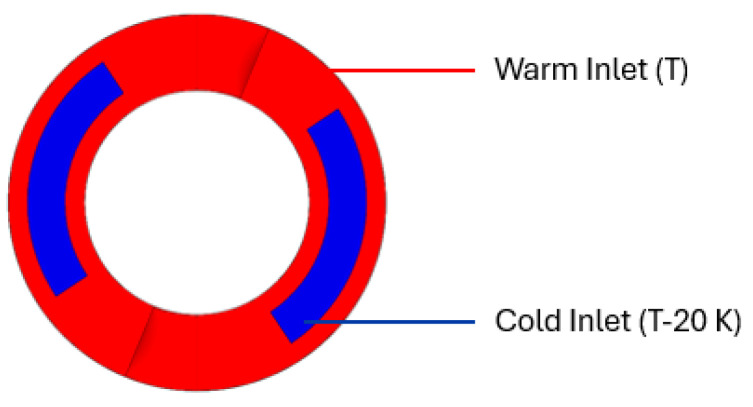
Inlet surfaces with different temperatures.

For the simulative investigations, software packages from Ansys Inc, Canonsburg (PA), United States, Version 2023 R1, were used for building the geometries with CAD (Ansys Fluent 2023 R1) in Ansys SpaceClaim, meshing the flow channels in Fluent Meshing, and simulating the flow with CFD in Ansys Fluent. For building up the simulations and fitting them to reality, the experimental investigations of screw B from [21] were used. The simulated flow channel of each screw had a total length of 270 mm, which is 4.5 D, with a 60 mm diameter, as shown in Figure 2. This was chosen because it is the length of one barrel element of the extruder used for experimental investigations and, therefore, holds 1/3 of the total offsets of a screw. It depicts the middle part of the extruder. Inlets and outlets were defined by a given, material-dependent pressure and, additionally, two different temperatures at the inlet. The two different parts of the inlet shown in Figure 3 differ by 20 K in temperature and should help to depict the behavior of the cold core in the rubber extrusion in simulations.

In addition to that, further assumptions were made regarding the simulation. This includes a constant temperature of the screw and barrel, wall adhesion, and pathlines, which are released only from the cold core to depict its behavior. The material behavior is modeled by a combination of the Carreau law and a WLF temperature shift in terms of viscosity, a pressure- and temperature-dependent user-defined function (UDF) for the density, and the temperature-dependent thermal conductivity and specific heat capacity. This model showed good accordance to the experimental trials for different screw and barrel temperatures as well as for different screw speeds.

Different alternative screw concepts on the basis of flight offsets as mixing elements were built up by geometric changes of the offset. Based on screw B, the total length of 18.3 D with a 60 mm diameter, the channel depth of 12 mm, the flight width of 5 mm, and the two flights with 7 mm flight flank radius were retained as the basic geometry. Therefore, the variation is limited to the four factors of flight pitch, circumferential and axial offset, and the number of offsets, which were varied in 5 steps, as shown in Table 4.

Based on this variation in the geometric factors, a central composite experimental design (CCD) with one center point was built, which results in 25 alternative screw geometries—shown in Table 5—that were considered for further investigations, as a full factorial design would have led to a disproportionately high simulation effort.

These alternative geometries were investigated with the CFD simulations with a screw speed of 30 rpm, a screw temperature of 353 K, and a barrel temperature of 343 K, which was shown to be a good setting for high throughput during the experimental investigations.

For the evaluation of the simulation results, there were three things to focus on. The first thing was the thermal properties of the rubber, which can be evaluated by, on the one hand, the maximum temperature difference in the outlet surface as a figure for the thermal homogeneity and, on the other hand, the average temperature as a higher temperature at the outlet, which is more energy efficient for constant screw and barrel temperatures as long as it remains below a critical material-dependent temperature—above which curing could occur. These values can be delivered by Ansys Fluent after the simulations. The material homogeneity was evaluated by a Delaunay triangulation of the tracer particles at the outlet surface, from which a value of the mixing quality can be calculated that can be found as part of Equation (1). The third relevant size for evaluating the results is the throughput that gets delivered by the screw, as this is a figure for the economic behavior of the screw. To rank the alternative screw concepts according to their performance, a key performance indicator was developed, which is shown in Equation (1), which consists of 45% of the thermal properties, 10% of material homogeneity, and 45% of the throughput, and is standardized to the best material-dependent value of each category, which can be either a minimum or maximum. The low consideration given to material homogeneity results from the fact that this is usually already achieved in the upstream mixing process on rolling mills or internal mixers.

(6) $$I_{sim}=0.45*\frac{{∆T}_{min,mat}}{∆T}*\frac{T_{ave,out}-T_{ave,in}}{T_{ave,out,max,mat}-T_{ave,in}}+0.1*\frac{{MQ}_{min,mat}}{MQ}+0.45*\frac{\dot{m}}{{\dot{m}}_{max,mat}}$$

where

∆T: Temperature difference at the outlet;
$T_{ave,out}$: Average temperature at the outlet;
$T_{ave,in}$: Average temperature at the inlet, constant;
$MQ=\frac{s}{\bar{x}}$: Mixing quality, calculated from triangles of the Delaunay triangulation;
s: Standard deviation of the triangles from the Delaunay triangulation;
$\bar{x}$: Mean value of the triangles from the Delaunay triangulation;
$\dot{m}$: Throughput.

**Figure 4:**
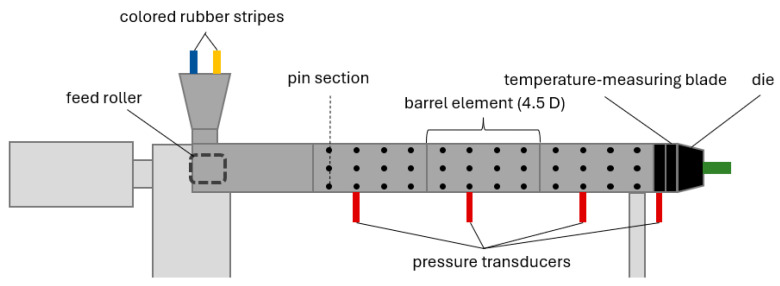
Experimental setup.

Based on the results from the simulations, three screws with the best performance according to Equation (1) were chosen and manufactured for experimental investigations on a 60 mm rubber extruder QSM 60k-12 D, Troester, Hanover (Germany). The modular extruder was used without pins and three barrel elements, leading to a total length of 18.3 D for all alternative screw concepts. For some comparative trials with the reference screw A, the extruder could also be equipped with pins on 12 pin levels with 6 pins each. A temperature-measuring blade is mounted at the screw tip, on which the temperature can be recorded at seven positions across the channel cross-section. A square-shaped throttling die with an opening of 17 × 17 mm is used. The configuration is depicted in Figure 4.

**Figure 5:**
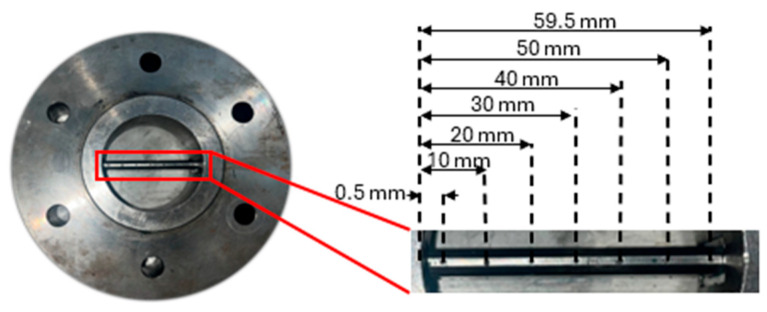
Measuring blade with 7 thermocouples.

During the experimental trials, the thermal homogeneity could be evaluated in accordance with the simulative trials due to the used measuring blade with 7 thermocouples at the screw tip, shown in Figure 5.

The throughput could also be measured during experimental trials. For the deviation of thermal homogeneity, the blue and yellow colored rubber was used, as it should result in a green extrudate at the die in the best-case scenario. Specimens were taken from the extrudate and vulcanized under high temperature, so that they could be cut into small slices. The slices were digitized by scanning and afterwards analyzed via ImageJ, version 1.54d, which is open-source Java software for image processing and analysis [22]. The area of the remaining blue and yellow material can be investigated using the color thresholding function in ImageJ. From those areas, the mixed part of the surface can be calculated by Equation (2):

(7) $$VF=A_{t}-(A_{y}+A_{b})$$

where

VF: Mixed area;
$A_{t}$: Total area;
$A_{y}$: Yellow area;
$A_{b}$: Blue area.

This was already used in [15,23], and shows itself to be a good method for quantifying the material homogeneity. In contrast to [15,23], the color thresholding values were changed, as those are empirical values that need to be set for each mixture separately, as already explained in [19].

As a result of that, the simulative key figure from Equation (1) needs some small adjustment to be used for experimental investigations as well, which results in Equation (3):

(8) $$I_{exp}=0.45*\frac{{∆T}_{min,mat}}{∆T}*\frac{T_{ave,out}-T_{ave,in}}{T_{ave,out,max,mat}-T_{ave,in}}+0.1*\frac{VF}{{VF}_{max,mat}}+0.45*\frac{\dot{m}}{{\dot{m}}_{max,mat}}$$

where

∆T: Temperature difference at the outlet;
$T_{ave,out}$: Average temperature at the outlet;
$T_{ave,in}$: Average temperature at the inlet, constant;
VF: Mixed area, evaluated with ImageJ;
$\dot{m}$: Throughput.

The experimental trials were carried out in two steps. In the first step, the trial point from the simulations was investigated experimentally to get a comparison between the simulations and reality. The screw speed for that point was 30 rpm, and the temperature profile is shown in Table 6.

In addition, the throughput of the screws was maximized by a rise in the screw speed while maintaining certain conditions. The maximum temperature during the trials was kept below that temperature to avoid scorching of the rubber compounds inside the extruder. Furthermore, the process had to stay stable so that no pulsation of the extrudate was detectable. In a final point, the trials were limited by the extruder, as the maximum screw speed was 190 rpm, and this should not be more than 90% of the maximum torque of the extruder, which means 3600 Nm, as the process should be kept safely reproducible.

## 3. Results

The results presented in the following are divided into the simulative results for all 25 new screw geometries and screw B, a comparison between experimental and simulative results, and experimental results with maximized throughput.

### 3.1. Simulative Results

**Figure 6:**
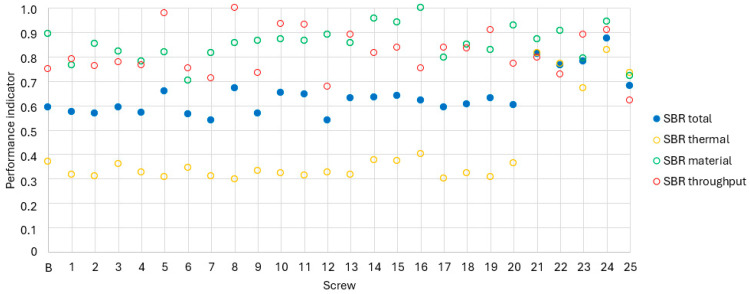
Simulative results of the SBR.

Figure 6 shows the results of simulations of alternative screw geometries for the extrusion of the SBR mixture.

The yellow data series presents the contribution of thermal homogeneity, represented by the first addend in Equation (8), to the overall performance index for the SBR compound. The normalized values of all investigated screw configurations (B and 1–25) exhibit only a narrow dispersion; however, the absolute level is low, ranging between 0.3 and 0.4. None of the screws show a significant deviation below this range, but screws 21 to 25 demonstrate markedly higher values than the remaining designs. The deviation from the mean is significant here, indicating that these screws achieve either a smaller outlet temperature difference or a higher mean outlet temperature. Examination of the raw simulation data confirms that both effects contribute to their improved thermal performance. However, no screw achieves the ideal value of 1, as the lowest temperature difference and highest material heating occur in different configurations.

The green data series illustrates the material homogeneity of the SBR compound, expressed as the ratio of each configuration’s MQ value to the maximum value obtained. All screws exhibit values between 0.7 and 1. Screw 16 attains the maximum index, while only a few other designs reach comparable performance. No consistent geometric characteristics can, however, be associated with superior material homogeneity. The lowest values are obtained for screw 25 (with the smallest pitch of 60 mm) and screw 6 (with 70 mm pitch and different offsets). Thus, no specific geometric feature can be identified that consistently leads to poor material homogeneity.

The normalized throughput index—derived as the ratio of each screw’s throughput to the highest value obtained—is shown in red, with normalized values ranging from 0.6 to 1. The maximum throughput (index = 1) is achieved by screw 8, closely followed by screw 5. Both possess a pitch of 90 mm, an axial offset of 5 mm, and nine offset planes, differing only in the circumferential offset (90 ° for screw 8, 30 ° for screw 5). Screws 10 and 11, likewise characterized by a 90 mm pitch, show similarly high values, while screw 24 with a 100 mm pitch reaches 0.91. The lowest throughput is again found for screw 25, which also exhibits the smallest pitch of 60 mm. These results confirm the general trend that increasing pitch leads to higher throughput under otherwise identical conditions.

The overall performance index, combining thermal and material homogeneity and throughput according to Equation (1), is depicted in blue. None of the screws achieve the ideal value of 1, indicating that no configuration is optimal across all criteria. Screw 24 yields the best overall compromise, characterized by a 100 mm pitch, twelve offset planes, and offsets of 10 mm (axial) and 60 (circumferential). Screws 21, 22, and 23 also perform above average. No screws show exceptionally low overall indices. In general, the SBR compound exhibits low normalized overall indices (around 0.6); however, this merely reflects a broader relative spread between higher- and lower-performing designs rather than inferior absolute performance.

Based on those results, screw 24 is chosen as the best screw for SBR, followed by screws 21 and 23, which will be produced and investigated in further experimental investigations.

### 3.2. Comparison Between Experimental and Simulative Results

The following three figures compare the experimental and simulative results for the SBR rubber mixtures. The experimental trials were carried out at 30 rpm with the temperature profile from Table 5, just as in the simulations. This led to pressures at the screw tip between 60 and 70 bar, with a fully closed throttle at the die. The pressure correlates with the throughput, as higher throughputs usually lead to higher back pressures at invariant temperature profiles [13].

**Figure 7:**
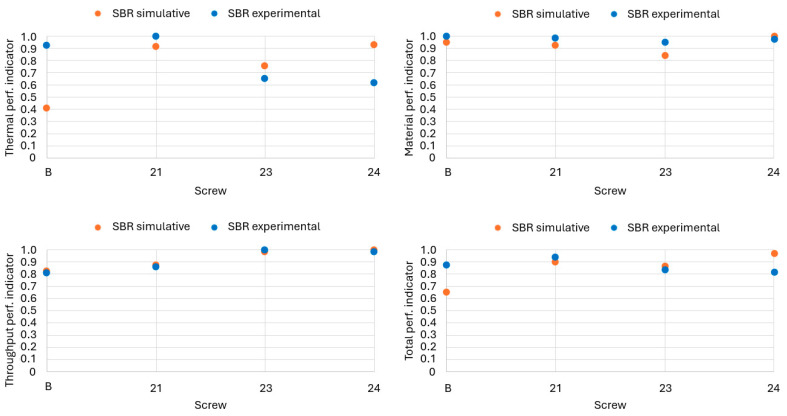
Comparison of simulative and experimental results for SBR.

Figure 7 shows discrepancies between simulated and experimental results for the SBR compound regarding the thermal figure, particularly for screws B and 24. The experimental data are not accurately reproduced by the simulations, resulting in a distortion of the relative ranking among screw designs. A comparison of the absolute outlet temperature differences and maximum outlet temperatures shows no meaningful correlation between the simulations and experiments, which might be caused by the shortened flow channel in the CFD simulations. In the experiments, screw 21 achieves the highest thermal homogeneity, whereas in the simulations, it ranks slightly below screw 24; the latter exhibited the poorest performance experimentally.

For the material figure, the experimental data show only minor differences between screw geometries, as all tested screws achieved a nearly similar degree of mixing at the screw tip. The simulations reproduce this trend, albeit with slightly greater variation. The best-performing screw in the experiments is B, while the simulations identify screw 24 as marginally superior; the experimental difference between these two designs amounts to approximately 3%. No direct correlation is observed between thermal and material homogeneity. The experimental data further confirm that low thermal homogeneity can coincide with high material homogeneity, implying that these properties are governed by distinct mechanisms.

The throughput figure shows an overall good agreement, with only minor deviations between predicted and measured results. The ranking of screws 23 and 24 differs. Experimentally, screw 23 produces the highest throughput, whereas simulations predict screw 24 to perform best. The difference between these two screws remains below 2% in both datasets. Although the absolute values differ substantially, as simulated throughput levels exceed experimental values, the normalized indices provide a solid qualitative and quantitative basis for comparing concepts, even if exact process prediction is not yet achievable.

The combined overall performance indicator, illustrated in Figure 7 on the bottom right, reflects these trends. Agreement between experiments and simulations is good for material homogeneity and throughput, while pronounced deviations persist for thermal homogeneity, resulting in noticeable differences in the total index, particularly for screws B and 24. Consequently, simulations in the preceding study identified screw 24 as optimal for SBR, whereas the experimental results under the investigated conditions (30 rpm, temperature profile 1) identified screw 21 as yielding the most favorable compromise between throughput and both thermal and material homogeneity.

### 3.3. Experimental Results with Maximized Throughput

The following chapter shows the results of the throughput maximization achieved by increasing the rotational speed. Table 7 shows the maximum speed achieved and the reason for the termination, which was always the maximum speed of the extruder, as the temperatures stayed below the critical temperature of 180 °C for all trial points. Afterwards, the screws are compared by the figures included in Equation (1). The pressure at the screw tip resulted in a wide range, between 70 and 110 bar, with a fully closed throttle at the die.

**Figure 8:**
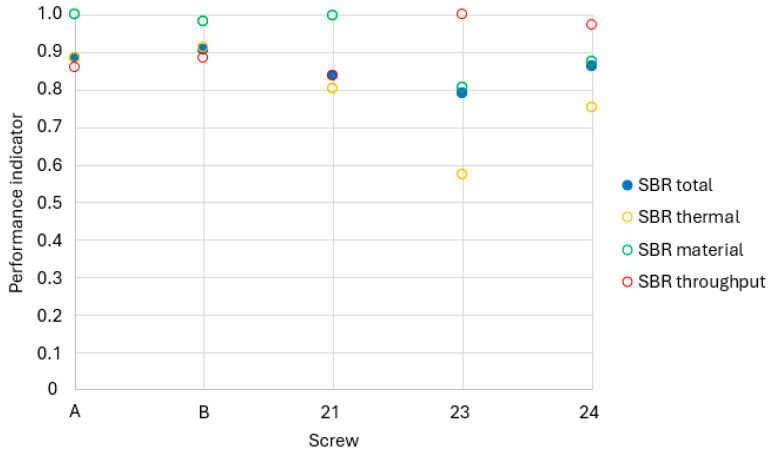
Results with maximized throughput for SBR.

Figure 8 shows the thermal homogeneity of the SBR compound for maximum throughput in yellow. The thermal results span a wide range from 0.57 to 0.91. The newly manufactured screws 21, 23, and 24 show lower values than the previously tested alternative screw B and the pinned reference screw A. The lowest thermal homogeneity is obtained for screw 23, while screw B achieves the best performance, slightly surpassing screw A. Analysis of the raw temperature data reveals pronounced differences of up to 8 °C in temperature gradients and 22 °C in mean outlet temperatures. Because both factors contribute equally to the normalized index, screws A and B benefit from their combination of low temperature differences and high mean temperatures. Screw B also demonstrates a smaller temperature gradient than the pinned design, confirming superior mixing efficiency, while screw 24 shows a lower overall average temperature, thus yielding a reduced index.

The material homogeneity of the SBR extrudates processed with different screw geometries is shown in green. The variation among screws is smaller than for thermal homogeneity, with indices ranging between 0.8 and 1.0. Screws A, B, and 21 exhibit nearly identical high values close to unity, with the pinned screw A achieving the maximum. Hence, while the pinned concept provides the best material homogeneity, two pin-free alternative screws deliver comparably good results. Screws 23 and 24 achieve lower values below 0.9 and visibly less uniform color distribution in cross-sections, though screw 23 still attains a mixed area of 0.78. The ranking of screws B, 21, 23, and 24 is consistent with that obtained at 30 rpm, confirming the validity of these results over a wide speed range up to 190 rpm.

The throughput figure at 190 rpm is marked in red. Despite the resulting identical operating conditions, the throughput variation is still recognizable. Screw 21, having the lowest index of 0.84, achieves 84% of the maximum throughput of screw 23. Comparison of throughput and material homogeneity diagrams reveals an inverse relationship: lower throughput is associated with improved mixing, suggesting that both screw geometry and extended residence time contribute to the enhanced material homogeneity. The ranking of screw performance agrees with both the experimental and simulated results obtained at 30 rpm.

The blue dots represent the total figure consisting of thermal and material homogeneity, as well as the throughput. Because thermal homogeneity exhibits the strongest differentiation, it dominates the overall index. Consequently, screw 23 receives the lowest total score of 0.79, while screw B achieves the highest with a value of 0.91. The pinned screw A ranks second, and the remaining alternative screws 21 and 24 fall in the midrange. Overall, the alternative screw concept, based on circumferential and axial flight offsets, provides a slightly improved overall performance in SBR extrusion compared to the conventional pinned design, confirming its potential as a viable substitute for the current state-of-the-art technology.

## 4. Discussion

Based on numerical simulations of various alternative screw concepts featuring circumferential and axial flight offsets, three screw geometries were selected for an SBR rubber compound. These screws were subsequently manufactured and experimentally tested. Although the simulations had been calibrated and validated using an experimentally characterized reference screw (screw B), noticeable deviations between simulated and experimental results were observed under identical operating conditions.

For the SBR compound, deviations are observed primarily in the thermal component of the overall performance index, which, owing to its weighting, also affects the total score. Those deviations might be caused by several effects, like imprecise material data, shortened computational domains, or rubber-related peculiarities. Nevertheless, the model provides good guidance on the correct design in terms of material homogeneity and throughput.

Since industrial rubber processing aims to achieve the highest possible throughput while maintaining the required product quality to ensure economic efficiency, additional experiments were conducted at maximum achievable throughput for the SBR rubber compound with all screws. In those experiments, screw B performed best in all tests, although it differs from the screw predicted as optimal by simulation. All alternative pinless screws, except screw 21, deliver higher throughputs than the pinned screw A.

In conclusion, two key findings can be drawn. First, the simulation of rubber extrusion processes using conventional CFD software is feasible and can serve as an effective decision-making tool for screw design. However, due to discrepancies observed for SBR thermal homogeneity, simulation models should be developed and validated on a broad experimental database before being used for predictive purposes. Second, the presented results demonstrate that screw concepts based on circumferential and axial flight offsets can yield performance comparable to, or even superior to, that of pinned extruders. Consequently, such simplified screw geometries can produce equivalent product quality at higher throughputs, thereby improving production efficiency.

The investigations did not reveal any significant disadvantages compared to screws secured with pins. However, one potential drawback is incomplete cleaning of the bore due to axial misalignment, which can become more significant as the axial misalignment increases and must therefore be considered.

For the future, these trials will be expanded to different rubber compounds to gather more information on the modeling of rubber extrusion and on optimum screw design for different rubber compounds. To improve the accuracy of the CFD simulations, it would be advisable to depict the whole screw rather than just a part of it. However, this decision must be made with consideration of the computing times.

## Figures and Tables

**Table 1:** Composition of the SBR compound.

Ingredient	Proportion of Recipe [phr]
SBR	90
NR	10
Plasticizer	42.5
Silica	65
Caolin	65
Coloring Agent	4
Minor Chemicals	19.7
Total	296.2

**Table 2:** Carreau parameters of the SBR compound.

*A*	261,004.7911 Pa·s
*B*	9.29142 s
*C*	0.71916
$T_{B}$	100 °C
$T_{S}$	−40.67841 °C

**Table 3:** Screw geometries of available screws at KTP.

Screw	Flight Pitch [mm]	Circumferential Offset [°]	Axial Offset [mm]	Number of Offsets [−]
A	80	54	12	12
B	80	22.5	25	12

**Table 4:** Variation in the screw geometry.

	Factor Levels				
	−−	−	0	+	++
**Flight pitch [mm]**	60	70	80	90	100
**Circumferential offset of the flight [°]**	0	30	60	90	120
**Axial offset of the flight [mm]**	0	5	10	15	20
**Number of offsets [−]**	6	9	12	15	18

**Table 5:** Twenty-five alternative screw geometries resulting from CCD experimental design.

Screw	Flight Pitch [mm]	Circumferential Offset [°]	Axial Offset [mm]	Number of Offsets [−]
1	70	30	5	9
2	70	90	5	9
3	70	30	15	9
4	70	30	5	15
5	90	30	5	9
6	70	90	15	9
7	70	90	5	15
8	90	90	5	9
9	70	30	15	15
10	90	30	15	9
11	90	30	5	15
12	70	90	15	15
13	90	90	15	9
14	90	90	5	15
15	90	30	15	15
16	90	90	15	15
17	80	60	10	12
18	80	120	10	12
19	80	0	10	12
20	80	60	20	12
21	80	60	0	12
22	80	60	10	18
23	80	60	10	6
24	100	60	10	12
25	60	60	10	12

**Table 6:** Temperature profile of experimental trials.

Temperature [°C]				
Feed Zone	Barrel 1 & 2	Barrel 3	Screw	Die
70	70	50	80	80

**Table 7:** Maximum screw speeds for the SBR mixture.

Screw	Maximum Speed [rpm]	Termination Criterion
A	190	Extruder maximum speed
B	190	Extruder maximum speed
Screw 21	190	Extruder maximum speed
Screw 23	190	Extruder maximum speed
Screw 24	190	Extruder maximum speed

## Data Availability

All data used in this article and further detailed information on the project can be provided by the authors. Please contact the corresponding author for further information.

## References

[1] Malaysian Rubber Council (2025). World Rubber Production, Consumption and Trade. https://www.myrubbercouncil.com/industry/world_production.php.

[2] Wirtschaftsverband der Deutschen Kautschukindustrie e.V (2025). Die Kautschukindustrie 2024|2025.

[3] Hallmann T. (2013). Untersuchung des Prozessverhaltens Neuartiger Einzugszonenkonzepte für Kautschukextruder. Ph.D. Thesis.

[4] Crowther B.G. (1998). Rubber Extrusion: Theory and Development.

[5] Röthemeyer F., Sommer F. (2013). Kautschuk Technologie.

[6] Abts G. (2007). Einführung in die Kautschukextrusion.

[7] Köster L., Perz H., Tsiwikis G. (2007). Praxis der Kautschukextrusion.

[8] Limper A., Barth P., Grajewski F. (1989). Technologie der Kautschukverarbeitung.

[9] Chen J., Dai P., Yao H., Chan T. (2011). Numerical Analysis of mixing performance of mixing section in pin-barrel single-screw extruder. J. Polym. Eng..

[10] Müllner H.W., Eberhardsteiner J., Fidi W. (2007). Rheological characterization of the die swell phenomenom of rubber compounds. Polym. Test..

[11] Schöppner V., Schmidt D., Schadomsky M. Investigations of High-Speed Rubber Extrusion with Consideration of Melt Homogeneity. Proceedings of the Deutsche Kautschuk Tagung (DKT 2018).

[12] Walter J.-U., Schöppner V., Schadomsky M. Bestimmung der Mischgüte bei Kautschukstiftextrudern auf Basis experimenteller und simulativer Methoden. Proceedings of the Technomer 2017.

[13] Brockhaus S. (2017). Theoretische und Experimentelle Untersuchungen zum Temperatur- und Durchsatzverhalten von Kautschukextrudern. Ph.D. Thesis.

[14] Yabushita Y., Brzoskowski R., White J.L., Najakima N. (1989). Flow of Rubber Compound in a Pin Barrel Screw Extruder. Int. Polym. Process..

[15] Rodger E.R., Roebuck H.S. (1976). Improved Economics through Scorch Control. J. Elastomers Plast..

[16] Schadomsky M. (2023). Experimentelle und Simulative Analyse der Mischwirkung in Einschneckenextrudern. Ph.D. Thesis.

[17] Sikora J.W. (2008). Increasing the efficiency of the extrusion process. Polym. Eng. Sci..

[18] Nijman G. (2000). Comparison between various screw-barrel-concepts for rubber extruders. Part 1: Presentation of the comparison-method. KGK Kautsch. Gummi Kunststoffe.

[19] Frank M. (2024). Simulationsgestützte Bestimmung der Optimierung der Mischgüte in der Einschneckenextrusion. Ph.D. Thesis.

[20] Spalding M.A., Sugden J.L., Welsh G.C. Fundamentals of foam sheet extrusion using a tandem extrusion line. Proceedings of the Antec 2023 Conference Proceedings.

[21] Schmidt L., Bruening F. Investigation of Alternative Pinless Screw Concepts for Rubber Extrusion. Proceedings of the Deutsche Kautschuk Tagung 2024 (DKT 2024).

[22] (1997). ImageJ–Image Processing and Analysis in Java.

[23] (2018). Simulative und Experimentelle Auslegung von Mischelementen Kaltgefütterter Kautschukextruder unter Besonderer Berücksichtigung der Viskoelastischen Materialmodellierung.

